# Robust and Precise Navigation and Obstacle Avoidance for Unmanned Ground Vehicle

**DOI:** 10.3390/s25144334

**Published:** 2025-07-11

**Authors:** Iván González-Hernández, Jonathan Flores, Sergio Salazar, Rogelio Lozano

**Affiliations:** Program of Aerial and Submarine Autonomous Navigation Systems, Department of Research and Multidisciplinary Studies, Center for Research and Advanced Studies, Mexico City 07360, Mexico; ivan.gonzalez@cinvestav.mx (I.G.-H.); jonathan.flores@cinvestav.mx (J.F.); sesalazar@cinvestav.mx (S.S.)

**Keywords:** unmanned ground vehicle, robust control, precise navigation

## Abstract

This paper presents a robust control strategy based on simplified second-order sliding mode for autonomous navigation and obstacle avoidance for an unmanned ground vehicle. The proposed control is implemented in a mini ground vehicle equipped with redundant inertial sensors for orientation, a global positioning system, and LiDAR sensors. The algorithm control avoids the derivative of the sliding surface. This provides a feasibility in real-time programming. In order to demonstrate stability in the system, the second method of the Lyapunov theory is used. In addition, the robustness of the proposed algorithm is verified through numerical simulations. Outdoor experimental tests are performed in order to validate the performance of the proposed control.

## 1. Introduction

Autonomous rover navigation from one location to another without human assistance has been extensively studied due to its wide range of applications, including data collection in remote or inaccessible areas, inspection and monitoring of hard-to-reach environments, and tracked locomotion for all-terrain navigation. Autonomous rovers play a crucial role in trajectory tracking and path following, as they are capable of navigating harsh environments such as nuclear power plants and sites requiring environmental monitoring, among others. These rovers and robots are equipped with specialized sensors that allow them to explore areas of interest, gather environmental data, and transmit this information back to operators to support decision-making processes.

Unmanned ground vehicles (UGVs) are increasingly prominent in the field of robotics. These systems typically have fewer actuators than degrees of freedom. They often include high-torque motors, a drive mechanism (engine or motor), wheels or treads for ground mobility, a battery for power supply, and solar panels for recharging. One of the key characteristics of autonomous vehicles is their ability to operate independently. Their capability to perform tasks without human intervention makes them a highly relevant and compelling area of study within robotics.

Nowadays, technology is continuously advancing toward greater automation, with minimal human interaction and increased ease of operation. Autonomous rovers are ground-based exploration robots designed to operate without human presence, equipped with varying levels of autonomous capabilities.

Currently, there are ongoing developments in the field of autonomous vehicles. Some research has focused on the development of semi-autonomous robots capable of operating on uneven terrain [1,2]. Other studies have aimed to implement autonomous capabilities in rescue robots [3,4]. Several other studies have explored the use of artificial neural networks in the domain of autonomous driving. Some approaches use vision-based systems for intersection detection [5,6,7], while others rely on neural networks that focus on key features of specific road types [8,9] or use anticipated road markings for navigation [10]. Some of these autonomous robots utilize various sensors, such as ultrasonic sensors, although these tend to offer lower precision.

In [11], the authors propose a robust and accurate localization scheme for unmanned ground vehicles (UGVs) operating in GPS-denied or weak-signal environments. Their approach employs multisensor fusion using LiDAR and an IMU through a Gaussian projection map technique.

In [12], the authors present a navigation method based on a Kalman Filter (KF), which integrates continuous vision, IMU mechanization, and geomagnetic measurements to achieve robust navigation performance. Although the algorithm uses two filters to improve robustness, this study is not focused on autonomous navigation.

Ref. [13] presents a practical setup that integrates two publicly available ROS packages with a recently released one to create a fully functional multi-session navigation system for ground vehicles.

In [14], the authors develop an adaptive and robust algorithm for GNSS and inertial navigation systems, incorporating data predictors based on a neural network architecture.

In [15], the authors present two robust controllers for a unicycle-type vehicle. The linear component of the controller design is based on the Lyapunov barrier function and the attractive ellipsoid method, incorporating input saturation, state constraints, and certain parameter uncertainties. The nonlinear component employs a sliding-mode integral controller that also considers state constraints.

Moreover, the authors in [16] use vision-based technology in autonomous vehicles and propose approaches for road safety under model perturbations and object occlusion based on deep learning models.

Controlling underactuated mechanisms presents significant challenges because techniques developed for fully actuated systems cannot be directly applied. These systems are not feedback-linearizable and exhibit nonholonomic constraints, as well as nonminimum phase characteristics [17].

In summary, from the articles cited above, it can be observed that in [13] the authors do not propose any control law for ground vehicle dynamics, while a control strategy based on a robust algorithm for GNSS and inertial navigation system has not been tested on autonomous navigation vehicles, as described in [14]. After that, other authors in [15] propose two robust controllers for a unicycle-type vehicle, but the experimental validation is conducted without incorporating obstacle avoidance. Meanwhile, the work presented in [11] does not offer a robust contribution to navigation under ambient or sensor noise disturbances. Moreover, in [12], although the authors present an algorithm that uses two filters to improve robustness, the study is not focused on autonomous navigation. In addition, [16] demonstrates that the use of a LIDAR sensor proves more cost-effective and accurate in short, safe navigation times with moving and static obstacles. Finally, in [17], although the authors present a class of systems that can be stabilized using continuous controllers, their control remains complex. One of the most common approaches for controlling underactuated systems is sliding mode control (SMC) based on a Lyapunov design. Hence, the objective of our work is to solve all these disadvantages that are presented in the references mentioned above by the different authors cited.

Therefore, the main contribution of this article is to implement a robust trajectory-tracking controller for an UGV system based on the first- and second-order sliding mode (1st-SM and 2nd-SM) control approaches to compensate all external disturbances that could affect the trajectory to be executed by the ground vehicle. The performance of the UGV system with the proposed controllers is evaluated through numerical simulations and real-time experiments. The contributions of this paper include presenting a chattering-free, asymptotic second-order sliding mode controller for the UGV system that does not require the derivative of the switching function. Additionally, one of the key advantages of this type of controller is its ability to reject external disturbances affecting the ground vehicle during the execution of any user-defined desired trajectory.

This paper is structured as follows: Section 2 presents the mathematical model and Section 3 presents the control strategy design for the unmanned ground vehicle. The numerical simulation results are shown in Section 4. Section 5 is devoted to the experimental platform, obstacle avoidance algorithms, and tree bark resin dot identification detection algorithms. Finally, discussion and conclusions are shared in Section 6.

## 2. Mathematical Model and Control

Unmanned ground vehicles (UGVs) are mechanically simpler than other autonomous vehicles, such as aerial vehicles or submarines. Their main advantage is that they do not rely on lift or buoyancy for movement. However, they face challenges such as wheel traction issues, irregular terrain, and obstacles on the path that can hinder the performance of their assigned tasks.

The performance of autonomous vehicles in executing tasks depends on the design of control algorithms that effectively respond to disturbances, ensuring accurate trajectory tracking in both numerical simulations and real-world environments.

### Dynamical Model

**Figure 1:**
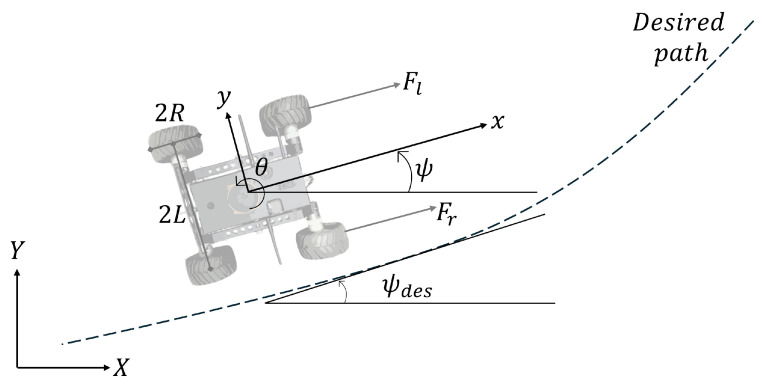
Unmanned ground vehicle top-view diagram.

Figure 1 shows the unmanned ground vehicle diagram and, according to [18], can be modeled in terms of the error with respect to the road as follows:

(1a) $$m\left(\ddot{x}-\dot{y}\;\dot{\psi }\right)=u\;cos\left(\theta +\psi \right)$$

(1b) $$m\left(\ddot{y}+\dot{x}\;\dot{\psi }\right)=u\;sin\left(\theta +\psi \right)$$

(1c) $$\;I_{z}\ddot{\psi }=\tau$$

where the control inputs are defined as $u=F_{r}+F_{l}$ and $\tau =LF_{l}-LF_{r}$ and the right and left forces are described as follows:

(2a) $$F_{r}=C_{r}\omega_{r}$$

(2b) $$F_{l}=C_{l}\omega_{l}$$

**Figure 2:**
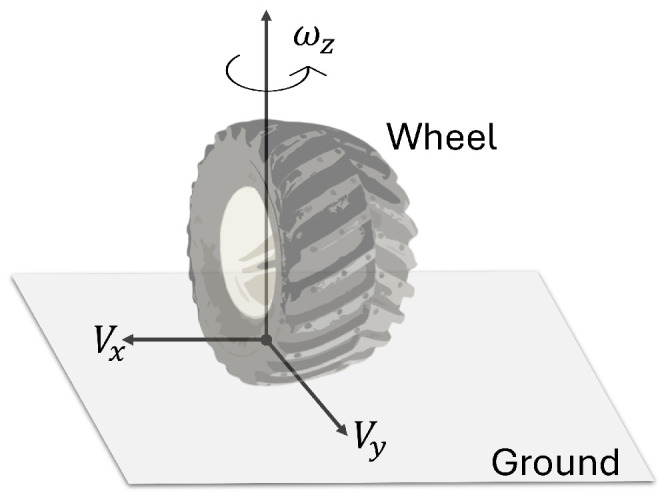
A diagram of the forces acting on the wheel in contact with the ground.

$C_{r}$ and $C_{l}$ are constants that represent a set of wheel constants, $\omega_{r}$ and $\omega_{l}$ are the wheel angular velocities (see Figure 2), *m* is the total mass, *v* is the longitudinal velocity, ψ is the angular position, *R* is the radio of the wheels, and *L* is the distance from the center of mass to the wheels. $F_{r}$ and $F_{l}$ are the left and right forces, respectively. $\tau_{r}$ is the right torque, $\tau_{l}$ is the left torque, and *J* is the inertia moment with respect to the center of mass.

Since the wheel is assumed to be a rigid element, it comes into contact with the ground at a single point that serves as the origin of the reference system shown in the previous figure, which is used to define the rotational speed that occurs in the wheels when the vehicle makes a turn on the road.

Based on the mathematical model, it is established that although the autonomous ground vehicle has four actuated wheels, it only has two PWM control signals. Therefore, the control signal u(t) consists of the left and right forces that control the vehicle’s movements in the x−y plane.

## 3. Control Algorithm Design

In this section, we present the robust control law for the unmanned ground vehicle (UGV) described by Equations (1a)–(1c). A second-order sliding mode control (SMC) algorithm is proposed due to its ability to reduce or eliminate the chattering effect inherent in first-order sliding mode controllers. Additionally, this robust control approach offers better performance in real-time applications by preventing wear on the actuators responsible for the movement of the UGV prototype.

Most second-order sliding mode (2nd-SM) control algorithms can achieve chattering-free control; however, they require the derivative of the sliding manifold $\sigma \left(t\right)$ for implementation. For instance, the 2nd-SM control, also known as the super-twisting algorithm [19], is designed as follows:

(3) $$\dot{u}\left(t\right)=-r_{1}sign\left(\dot{\sigma }\left(t\right)\right)-r_{2}sign\left(\sigma \left(t\right)\right)$$

In order to avoid a chattering-free SM, the control law is given by

(4) $$u\left(t\right)=-r_{1}\int_{0}^{t}sign\left(\dot{\sigma }\left(t\right)\right)dt-r_{2}\int_{0}^{t}sign\left(\sigma \left(t\right)\right)dt$$

where $r_{1}$ and $r_{2}$ are positive constants.

To avoid using the derivative of σ(t), it is possible to stabilize the UGV system given by the three dynamics of (1a)–(1c) by the 2nd-SM control with a 1st-SM control law [17,20,21,22]:

(5) $$\dot{u}\left(t\right)=-k_{2}sign\left(\sigma \left(t\right)\right),\;k_{2}>0$$

To improve the convergence time, a feedback control term is added to the above 1st-Sm control law as

(6) $$\dot{u}\left(t\right)=-k_{1}\dot{\sigma }\left(t\right)-k_{2}sign\left(\sigma \left(t\right)\right)$$

Therefore, by integrating the previous control law, a chattering-free 2nd-SM control without using the derivative of the switching function σ(t) is proposed as

(7) $$u\left(t\right)=-k_{1}\sigma \left(t\right)-k_{2}\int_{0}^{t}sign\left(\sigma \left(t\right)\right)dt$$

Here, $k_{1}$ and $k_{2}$ are positive constants to be designed such that the UGV is stabilized asymptotically.

## 4. Numerical Simulations

In the simulation, the dynamics of the systems described by Equations (1a)–(1c) are considered to observe the performances of the control strategies proposed in Section 3 for the UGV vehicle with the following two cases:

- 1st-SM control law;
- 2nd-SM control law (also called the super-twisting algorithm).

For both cases of numerical simulations, it is assumed that the initial conditions ($x_{0}$, $y_{0}$, $\psi_{0}$) are equal to (1 m, 1 m, 0.9 rad) and that the control input u(t) is bounded with

(8) $$\left|u\left(t\right)\right|<30$$

The switching functions of the robust control law are designed for each dynamic as follows:

(9) $$\begin{array}{cc} \sigma_{x}\left(t\right) & =5x\left(t\right)+\dot{x}\left(t\right) \end{array}$$

(10) $$\begin{array}{cc} \sigma_{y}\left(t\right) & =4y\left(t\right)+\dot{y}\left(t\right) \end{array}$$

(11) $$\begin{array}{cc} \sigma_{\psi }\left(t\right) & =9\psi \left(t\right)+\dot{\psi }\left(t\right) \end{array}$$

such that the UGV system on the sliding mode σ(t)=0 is asymptotically stable.

**Figure 3:**
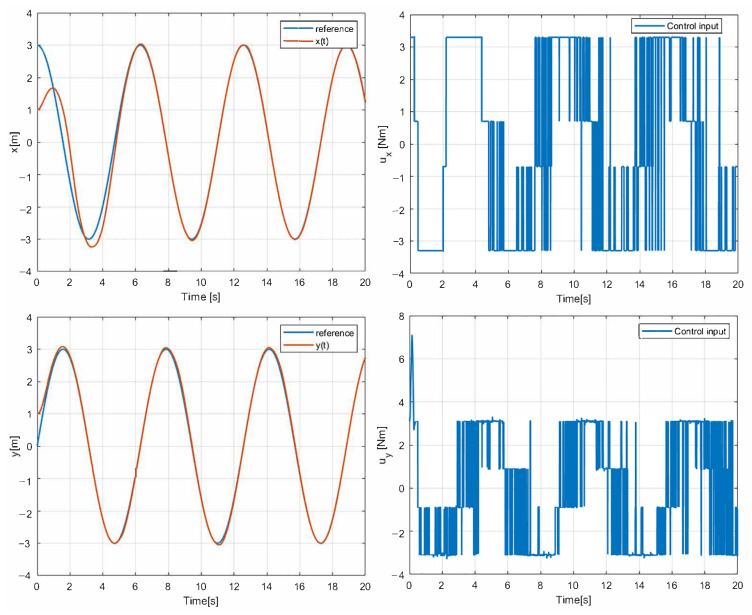
Simulation result with 1st-SM control.

**Figure 4:**
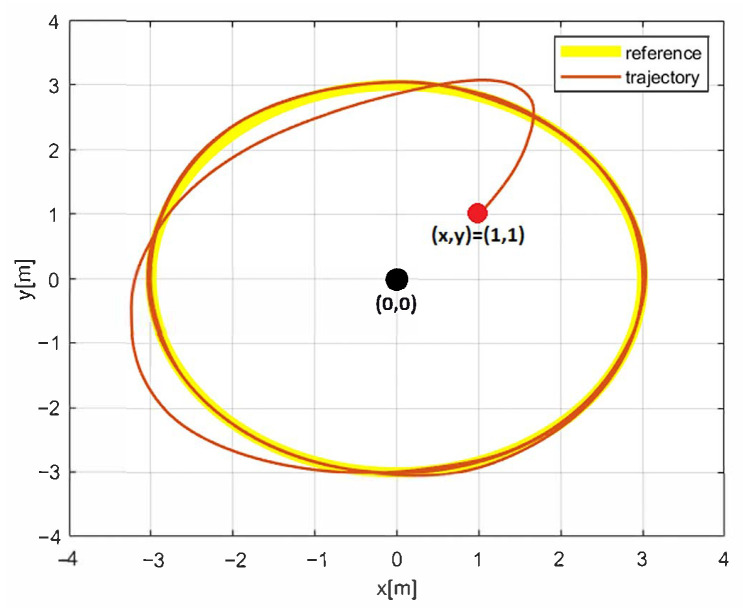
The simulation result of the desired trajectory using 1st-SM control.

**Figure 5:**
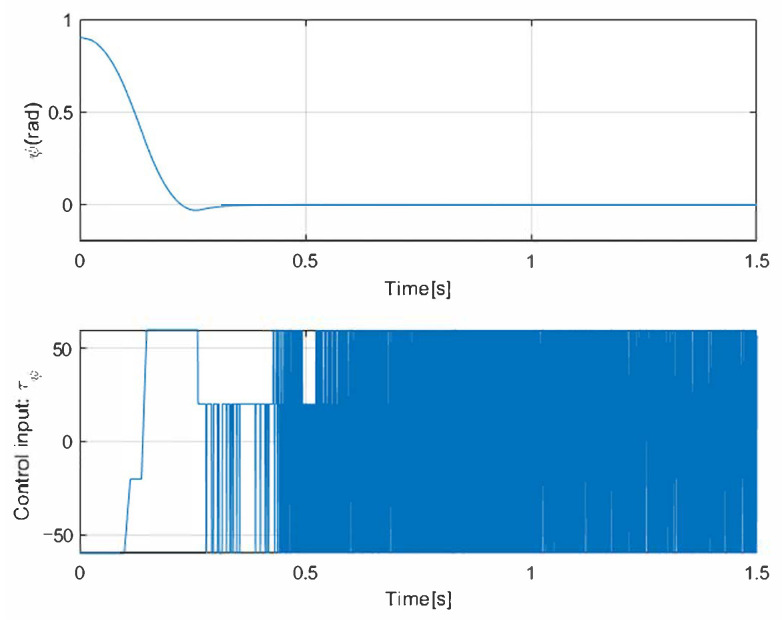
The simulation result of the ψ-angle using 1st-SM control.

At first, a 1st-SM control law u(t)=−30sign(σ(t)) is implemented in the simulation using a circular path as a reference. In addition, Gaussian noise is added to these simulations to verify the robustness of the navigation. The two main parameters of the noise added in the simulation to observe the behavior of the vehicle in the face of possible external disturbances are the mean with a value of 0 and the variance with a value of 1. The results given in Figure 3 show that the states of position (*x*, *y*) converge in finite time with the 1st-SM control law. The control input designed above is asymptotically stable. In this case, however, the chattering phenomena can be observed as the 1st-SM control input u(t) is switched among ±30 at a high frequency. Figure 4 and Figure 5 correspond to the evolution of the circular trajectory tracking for the UGV vehicle and the angle error ψ that represents the vehicle deviation from the path to follow. It can be seen that the vehicle reaches the desired reference, but a bias is preserved that is not desirable.

**Figure 6:**
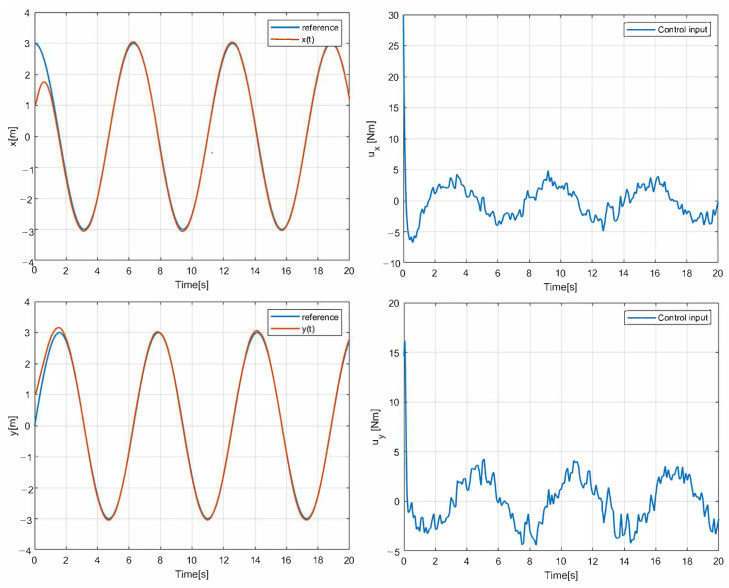
The simulation result with chattering-free 2nd-SM control.

**Figure 7:**
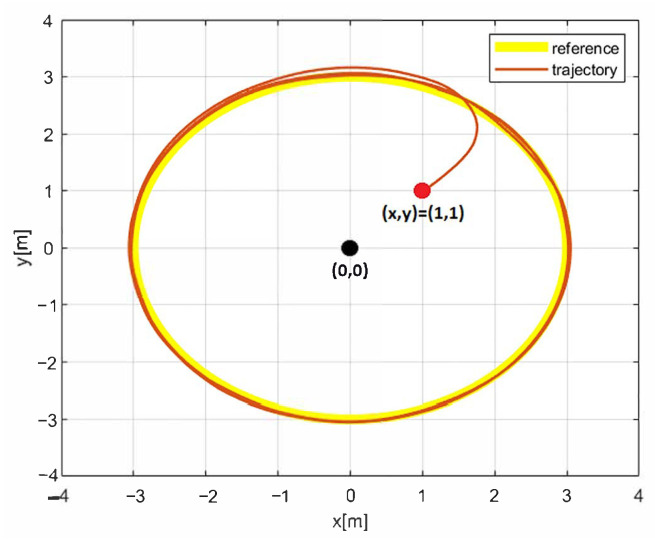
The simulation result of the desired trajectory using 2nd-SM control.

**Figure 8:**
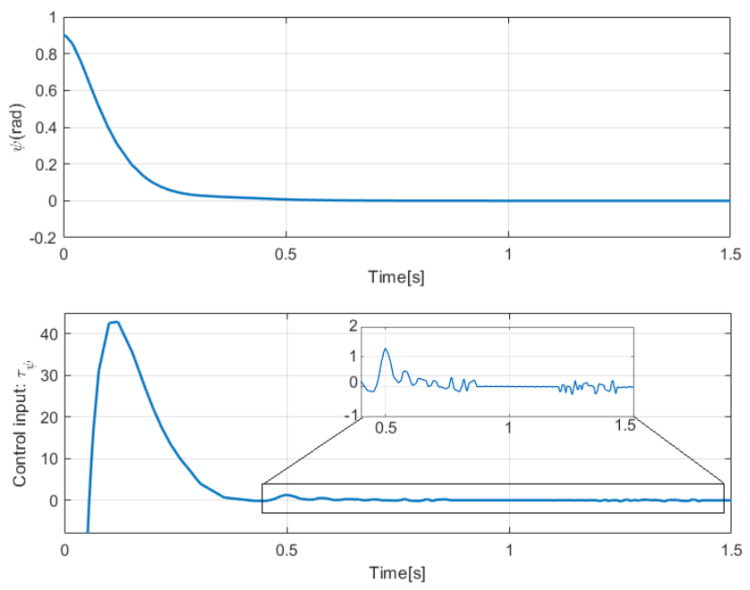
Simulation result of the ψ-angle using 2nd-SM control.

The simulation results with the proposed 2nd-SM control law (7) are shown in Figure 6, Figure 7 and Figure 8, where the control gains are shown in Table 1. It is clear that second-order SM control allows the UGV vehicle to adequately reach the desired circular path asymptotically, the state variables x(t) and y(t) converge to the references of the *sine* and *cosine* components of the desired circular path asymptotically, and u(t) is smooth in comparison with the 1st-SM control law shown in Figure 3. Thus, the system described by (1a)–(1c) with the chattering-free 2nd-SM control law (7) is asymptotically stable without a chattering effect. It is also confirmed in the simulation that the system cannot be stabilized by the integrated 1st-SM control law, i.e., the proposed robust control manages to stabilize the UGV in the desired reference of the circular trajectory. Obviously, the 2nd-SM control algorithm ensures the finite time convergence of states x(t), y(t) and ψ(t) to the references given, as shown Figure 6 and Figure 8, respectively.

The effectiveness of the 2nd-SM control algorithm applied to the UGV model is demonstrated through a series of numerical simulations, which show a significant reduction in the chattering effect in the control input used to reject external disturbances affecting the vehicle. These results are further confirmed in the following section presenting the experimental results.

## 5. Experimental Results

**Figure 9:**
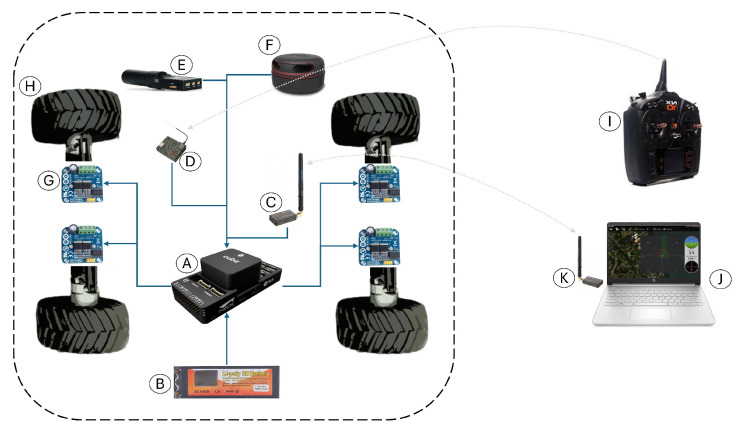
Unmanned fround vehicle component diagram.

The unmanned ground vehicle (UGV) experimental platform has four actuated wheels and does not have servomotors. The forward direction is controlled by the difference in speed of its right and left wheels. A Rocker Bogie suspension system is used to prevent overturning and to cushion mild uneven terrain. Figure 9 shows the interaction diagram of the electronic components, where sensors transmit information about the UGV and its surroundings and, after processing it, command the actuators to control navigation. Table 2 lists and names these components, as well as their general functions.

**Figure 10:**
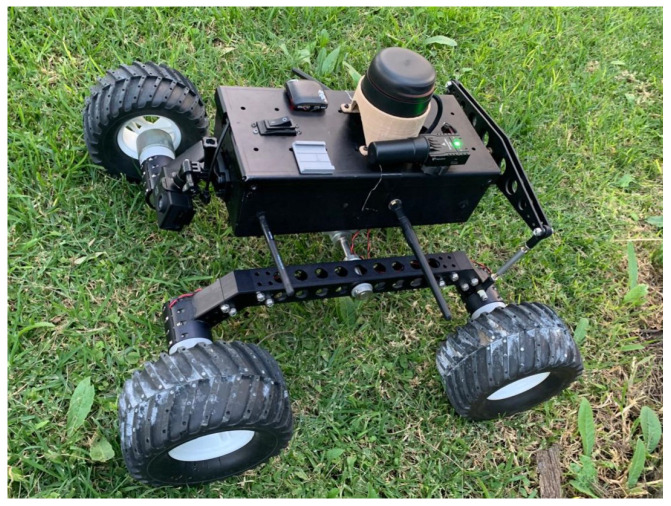
Unmanned ground vehicle platform.

The Controller board was used, which was compatible with a open-source firmware due to its support for embedded and peripheral precision sensors. Control algorithms developed in Section 2 were developed and simulated in Matlab and Simulink in Section 3 and implemented in the controller board. A Global Navigation Satellite System (GNSS) was used for navigation, using differential devices to achieve centimeter-level accuracy for autonomous navigation of the unmanned ground vehicle. Some piles of cut grass were used to induce perturbations to the UGV in circular trajectory tracking. Figure 10 shows the servoless four-wheeled ground vehicle used in this research, which used the differential-drive forces of its left and right wheels for its displacement.

**Figure 11:**
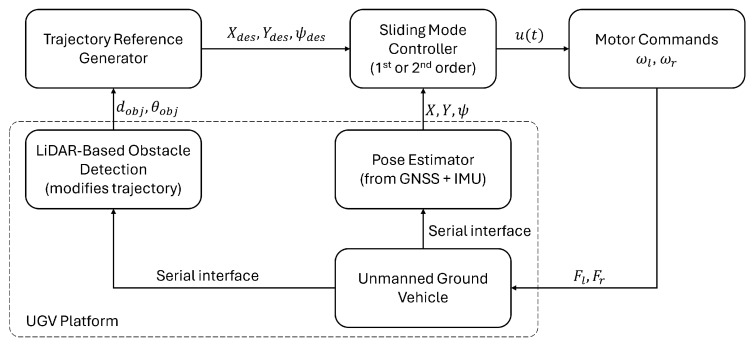
Overall control architecture.

Figure 11 illustrates the overall control architecture implemented for the unmanned ground vehicle (UGV). The system begins with a trajectory reference generator, which defines the desired path based on mission way-points. This trajectory can be dynamically modified by a LiDAR-based obstacle avoidance module, which detects obstacles in the environment and adjusts the path accordingly to ensure collision-free navigation.

The reference trajectory is then compared to the current pose of the UGV (position and orientation), and the sliding mode controller—either first- or second-order—calculates the appropriate control actions. These actions are converted into motor commands for the left and right wheels ($\omega_{l}$, $\omega_{r}$), which drive the physical UGV platform.

To close the control loop, the Pose Estimator, which fuses data from a GNSS and IMU, provides updated state information to the controller, enabling real-time correction and stability. This cascaded control scheme ensures a robust trajectory tracking while maintaining obstacle avoidance and disturbance rejection capabilities.

### 5.1. Position Control

**Figure 12:**
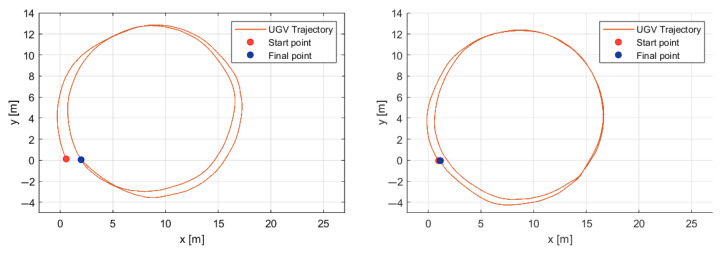
Circular path tracking using first-order (**left**) and second-order (**right**) sliding mode control techniques.

The experiments consist of following a circular path twice around the same point (4, 8.5) with a radius of 8.5 m. The UGV encounters two grass piles near the points (5, 12) and (15, −2), which induce perturbations in the path tracking. The second-order sliding mode control algorithm preserves the same start and end points of the path more accurately despite the perturbations induced by the grass piles. Figure 12 shows the start points, paths, and end points of the UGV following a circular path twice using the first- and second-order sliding mode control algorithms on the left and right side, respectively.

### 5.2. Obstacle Avoidance

**Figure 13:**
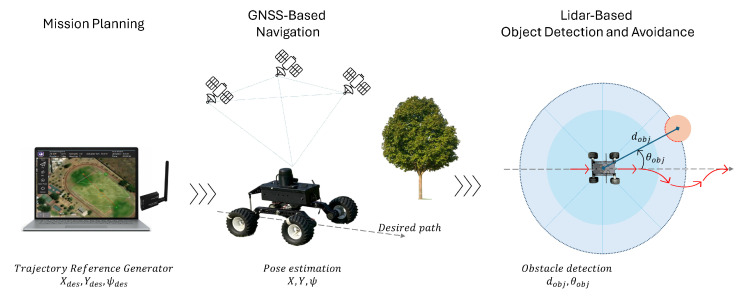
UGV navigation and object avoidance concept.

The Unmanned ground vehicle is equipped with the RPLiDAR A2 full-range omnidirectional laser scanner, which senses distances up to 10 m around the vehicle in the horizontal plane. This laser scanner detects obstacles such as clumps of grass along the path during autonomous missions. The obstacle avoidance algorithm then selects an open path to continue the mission, allowing the vehicle to follow an alternate route to the final destination. Figure 13 shows robust and precise navigation for the UGV that involves the following stages:

1. Mission Planning: The trajectory is defined at the ground station and transmitted to the vehicle. This trajectory is based on the vehicle’s absolute position and sequential tracking of way-point using a high-precision outdoor GNSS positioning system.
2. GNSS -Based Navigation: The path-following algorithm is robust to terrain irregularities. The vehicle follows the navigation route using an embedded magnetometer into the autopilot, and the trajectory is regulated by varying the speed of the left and right wheels.
3. Object detection: A LiDAR-based omnidirectional distance sensor emits light beams to generate a point cloud of obstacles along the trajectory. Obstacles are detected, and the distance and angle of the object relative to the UGV are determined ($d_{obj}$, $\theta_{obj}$).
4. Obstacle avoidance: When an obstacle is detected during UGV trajectory tracking, the evasion algorithm calculates an alternative course that allows the vehicle to evade the obstacle by turning right or left depending on the clearest path (toward where the lidar sensor does not detect any obstacles). When the vehicle no longer detects the obstacle, it resumes its trajectory toward the next waypoint.

Due to the point cloud dispersion (with a density of 4000 at 10 Hz), obstacle detection is effective when the obstacle has a radius greater than 4 cm; this detection improves as the object approaches. Before the autonomous mission, a safety radius of 1 m is established around the vehicle; a greater range would not allow the vehicle to pass between two nearby obstacles. Signal processing from the global and local positioning sensors, as well as the inertial sensors, enables robust and precise navigation.

A companion computer is not necessary for the navigation system’s obstacle detection and avoidance due to its low computational cost, as it does not require map reconstruction or memory to store obstacle data. The obstacle avoidance algorithm calculates possible curved secondary paths as alternate route options for the vehicle, which then orients itself back to the assigned path. The vehicle detects objects along its route, with an evasion radius assigned both to the vehicle and to the target obstacle.

**Figure 14:**
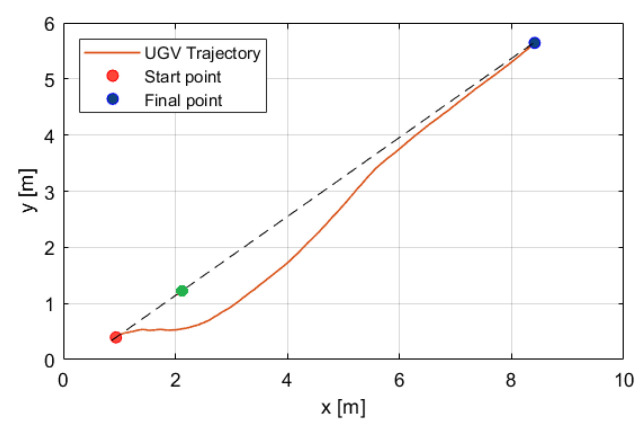
Object (green point) avoiding with a radius of 1.5 m during the UGV autonomous mission.

Figure 14 illustrates the avoidance of an object (green point) during straight path as follows: The vehicle begins avoidance (red point) 1.5 m before the obstacle and assigns a 1-meter radius around it. Afterwards, the UGV continues toward the end point of the path (blue point). The dotted line represents the straight path between the start and final points.

**Figure 15:**
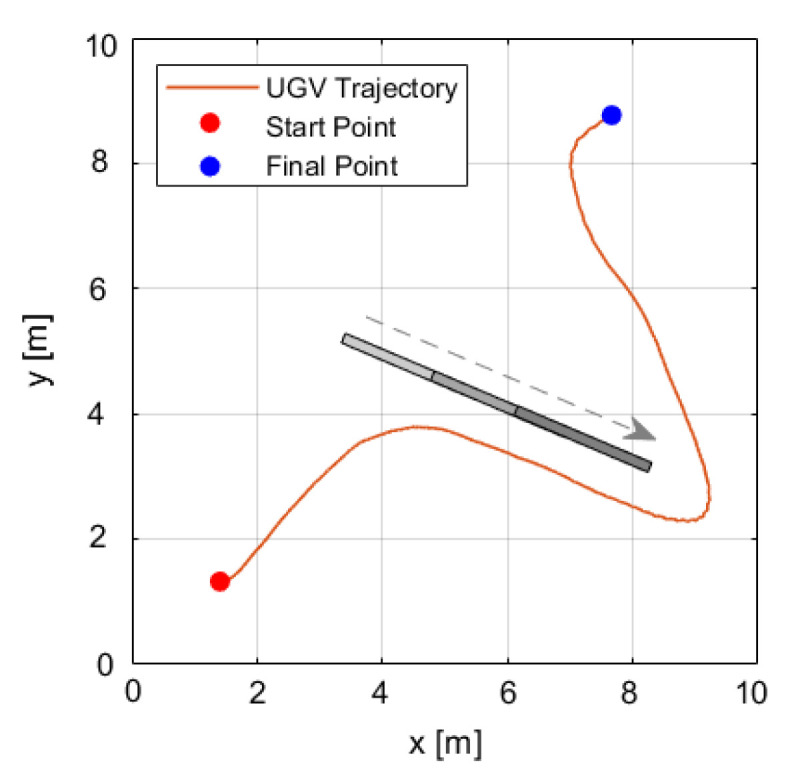
Moving obstacle (gray box) avoidance during the UGV autonomous mission.

Figure 15 shows the trajectory of the unmanned ground vehicle (UGV) from its starting point to its end point. During its path, the UGV encounters a moving obstacle traveling parallel to it at the same speed. When the obstacle stops, the UGV continues its mission by navigating around it, as if bypassing a wall. The vehicle maintains an approximate distance of 0.5 m from the obstacle until it safely passes.

Table 3 shows the performances of the first- and second-order sliding mode controllers in terms of the standard deviation of position and velocity in the autonomous mission.

## 6. Conclusions and Discussion

A robust control technique based on a chattering-free asymptotic second-order sliding mode (2nd-SM) controller has been presented for autonomous rover vehicle navigation. This approach does not require the derivative of the switching function. A comparison was made between the proposed controller and the classical first-order sliding mode controller.

The simulation results demonstrated the good performance of the proposed sliding mode control, allowing the ground vehicle to be stabilized on the desired circular trajectory within a 0.1 m accuracy. Additionally, the typical chattering effect was significantly reduced in the control inputs, facilitating easier real-world implementation. This improvement was due to the pre-feedback control term included in the control law, which ensured that the necessary condition for sliding mode control was locally reached, guaranteeing asymptotic stability.

Experimental results with the UGV were achieved in several scenarios, including position control and obstacle avoidance. In the position control experiments, the tracking error was around 0.08 m, while the error during obstacle avoidance was approximately 0.07 m. 

## Figures and Tables

**Table 1:** Unmanned ground vehicle simulation parameters.

Parameter	Value	Parameter	Value	Parameter	Value
*m*	1 kg	Radious	3 m	$k_{2_{x}}$	1
*R*	0.15 m	x(0)	1 m	$k_{1_{y}}$	5
*L*	0.5 m	y(0)	1 m	$k_{2_{y}}$	1
$x_{d}$	3 m	ψ(0)	0.9 rad	$k_{1_{\psi }}$	10
$y_{d}$	3 m	$k_{1_{x}}$	5	$k_{2_{\psi }}$	8

**Table 2:** Unmanned ground vehicle list of components.

Part	Name	Function
A	Autopilot	Sensor signal processing and actuator control.
B	Battery	Autopilot and actuator power supply.
C	Data Radio	Onboard telemetry transmitter and receiver.
D	RC Receiver	Radio control receiver.
E	GNSS Antenna	Global Navigation Satellite System Antenna.
F	Rangefinder	LiDAR-based omnidirectional distance sensor.
G	Motor Driver	Motor speed controller.
H	Motor and Wheel	Actuator to control position and orientation.
I	RC Transmitter	Radio Control Transmitter.
J	Ground Control Station	Ground-based system used to monitor and control.
K	Data Radio	Ground control station telemetry transmitter and receiver.

**Table 3:** The standard error of the mean.

Parameter	Value	Units
1st-Order Sliding Mode Position standard deviation	0.13	m
2nd-Order Sliding Mode Position standard deviation	0.08	m
1st-Order Sliding Mode Velocity standard deviation	0.09	m/seg.
2nd-Order Sliding Mode Velocity standard deviation	0.07	m/seg.

## Data Availability

The original contributions presented in this study are included in the article. Further inquiries can be directed to the corresponding author.

## Abbreviations

The following abbreviations are used in this manuscript:

UAV	Unmanned Aerial Vehicle
UGV	Unmanned Ground Vehicle
IMU	Inertial Measurement Unit
KF	Kalman Filter
PWM	Pulse Wide Modulation
I2C	Inter Integrated Circuit
UART	Universal Asynchronous Receiver-Transmitter
CAN	Controller Area Network
SPI	Serial Peripheral Interface
ADC	Analog-to-Digital Converter
LiDAR	Light Detection and Ranging
ROS	Robot Operating System
GPS	Global Positioning System
GNSS	Global Navigation Satellite System
PD	Proportional Derivative
